# Microplastic surface retention and mobility on hiking trails

**DOI:** 10.1007/s11356-023-25635-z

**Published:** 2023-01-31

**Authors:** Nicola Ann Forster, Susan Caroline Wilson, Matthew Kevin Tighe

**Affiliations:** School of Environmental and Rural Science, University of New England, Armidale, New South Wales 2351 Australia

**Keywords:** Rainfall simulation, Microplastic movement, Soil surface, Landscape function analysis, Erosion

## Abstract

Hiking and trail running are a source of microplastic (MP) pollution on recreational trails in wilderness and conservation areas; however, the fate of MPs deposited on trails is poorly understood as MP mobility on such surfaces has not yet been examined. In this study, we simulated heavy rainfall (100 mm/h) on trail surfaces with existing MP pollution (in situ MPs) and spiked with 99 ± 2 rubber MPs (100–940 μm). Runoff was collected for 15 min and spiked and in situ MPs were quantified. Hydrological, erosional and microplastic responses were evaluated in relation to slope, bulk density, soil moisture and surface condition indicators, including amounts and types of surface cover and soil physical attributes. The MPs were largely immobile, with 85–100% of spiked MPs retained on trail surfaces. In situ MPs were detected in the trail runoff, with the majority being polyurethane, polypropylene and polyester. Microplastic movement was primarily influenced by hydrological effects, and analysis indicated the main explanatory variable was total runoff volume, followed by soil slaking. Trail sections with at least 15% herbaceous cover or a layer of loose alluvium had higher MP retention. Areas of resource accrual may be preferentially enriched, suggesting MPs from outdoor recreation may be concentrated on and adjacent to recreational trails. Microplastics deposited on trails may have long term implications for biodiversity and ecosystem functioning in wilderness and conservation areas, particularly around the trail corridor.

**Supplementary Information:**

The online version contains supplementary material available at 10.1007/s11356-023-25635-z.

## Introduction

Outdoor recreation (e.g. hiking, trail running, mountain biking and climbing) is increasingly popular worldwide and may be a source of microplastic (MP) pollution in wilderness and conservation areas (Forster, Tighe et al. 2020, Forster, Tighe et al. 2022). Rubber, polyethylene, polystyrene, polypropylene, polyester and polyurethane particles detected in protected and wilderness areas have been attributed to the abrasion and fragmentation of clothing, shoes, mountain bike tires and litter (Barrows, Christiansen et al. 2018, Parolini, De Felice et al. 2021). Microplastic deposition is thought to be concentrated on recreational trails; however, preliminary studies suggest MPs may move from trails to adjacent areas (Ambrosini, Azzoni et al. 2019, Napper, Davies et al. 2020).

Very little is known about lateral MP mobility on soil surfaces and the potential implications for the spatial redistribution of MPs in protected areas. Soil on trail surfaces is typically compacted due to foot traffic, which may limit downwards migration of MPs and promote lateral movement (Li, Song et al. 2020). Soil organisms and wind have been found to transport MPs laterally on soil surfaces (Huerta Lwanga, Gertsen et al. 2017, Maaß, Daphi et al. 2017, Rezaei, Riksen et al. 2019). Preliminary research indicates rainfall also moves MPs laterally; however, there is significant variation in the distance travelled depending on MP size and shape, rainfall intensity and surface conditions (Laermanns, Lehmann et al. 2021, Zhang, Chen et al. 2022). Microplastic mobility may be extremely diverse on recreational trails, where slope, compaction, litter, vegetation, surface roughness and soil texture can be highly variable (Liddle 1997, Sutherland, Bussen et al. 2001, Olive and Marion 2009).

Recreational trails frequently traverse ecologically significant areas characterised by high biodiversity and endangered or unique plant and animal species (Pickering and Hill 2007, Siikamäki, Kangas et al. 2015). Microplastics that move off-trail may impact these areas through direct and indirect effects on soil organisms, microbiology, soil chemistry and soil structure (Bandow, Will et al. 2017, de Souza Machado, Lau et al. 2018, Chen, Liu et al. 2020, Kim, Waldman et al. 2020, Pflugmacher, Huttunen et al. 2020, Prüst, Meijer et al. 2020). Ecosystem functioning may be altered as long-term exposure to MPs has been shown to influence soil-water dynamics, soil respiration, nutrient cycles, litter decomposition and plant growth and development (de Souza Machado, Lau et al. 2019, Lozano and Rillig 2020, Yi, Zhou et al. 2020, Leifheit, Kissener et al. 2021, Zhao, Lozano et al. 2021). Thus, MP impacts on the soil environment may be greater in sections with increased MP pollution due to higher deposition and/or retention.

The objective of this study was to investigate the relationship between MP mobility, trail topography and hydrological and erosional responses to rainfall. This was done using a combination of rainfall simulation and trail surface characterisation, including the use of a rapid soil surface assessment approach (Tongway and Hindley 2004).

## Methods

### Study location

**Fig. 1:**
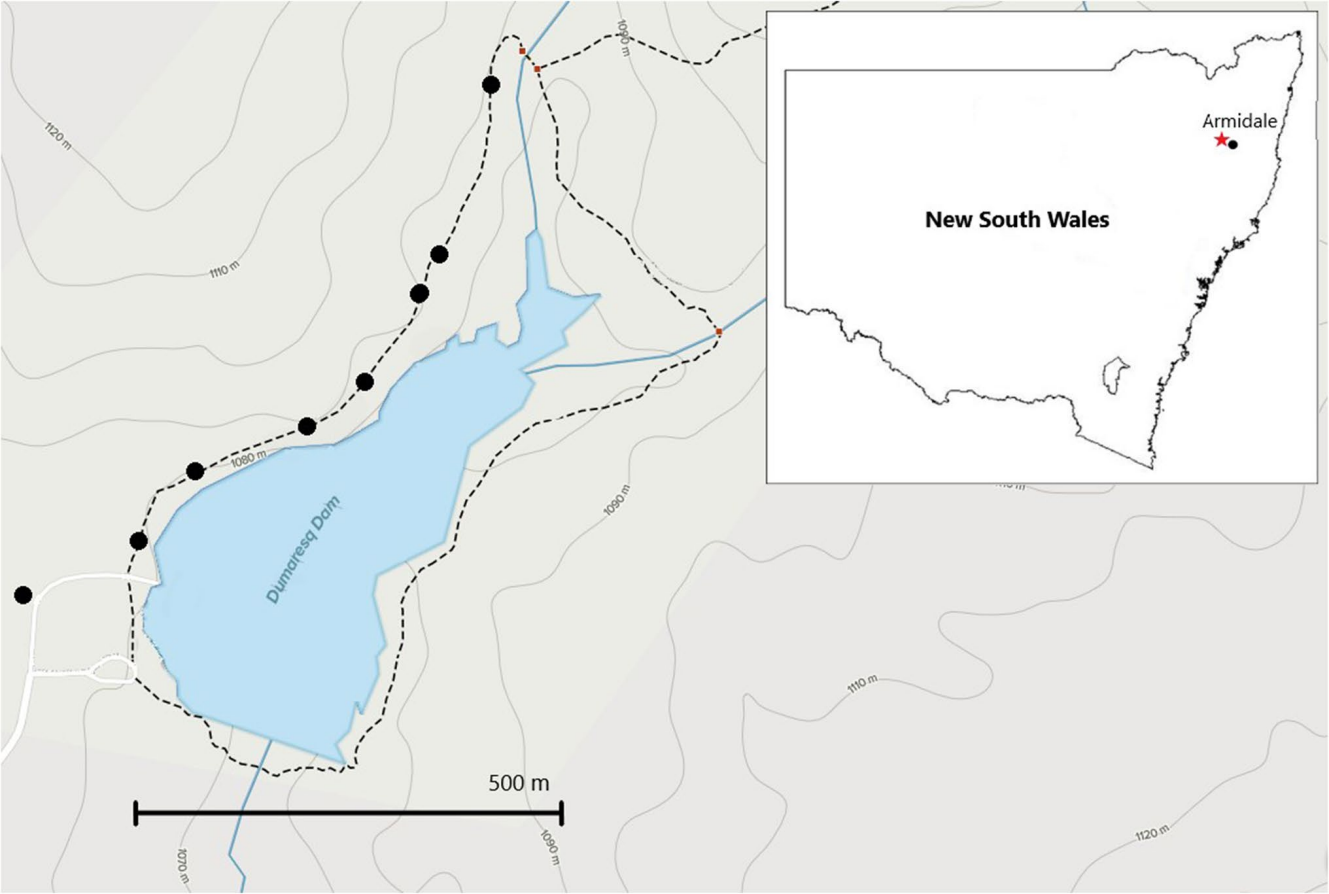
Walking and running trail in Dumaresq Dam Reserve, located near Armidale, NSW, Australia. Black markers represent plot locations. Map modified from AllTrails (AllTrails 2022). Inset is map showing location of Armidale within the state of New South Wales

The study area was a walking and running trail in Dumaresq Dam Reserve (30°25′49″S, 151°35′58″E) near Armidale, New South Wales, Australia (Fig. 1). This site is maintained by the Armidale Regional Council and is popular for bushwalking, running, kayaking and fishing. There is an annual trail running event with up to 400 participants (Armidale Athletics Club Inc 2022).

**Fig. 2:**
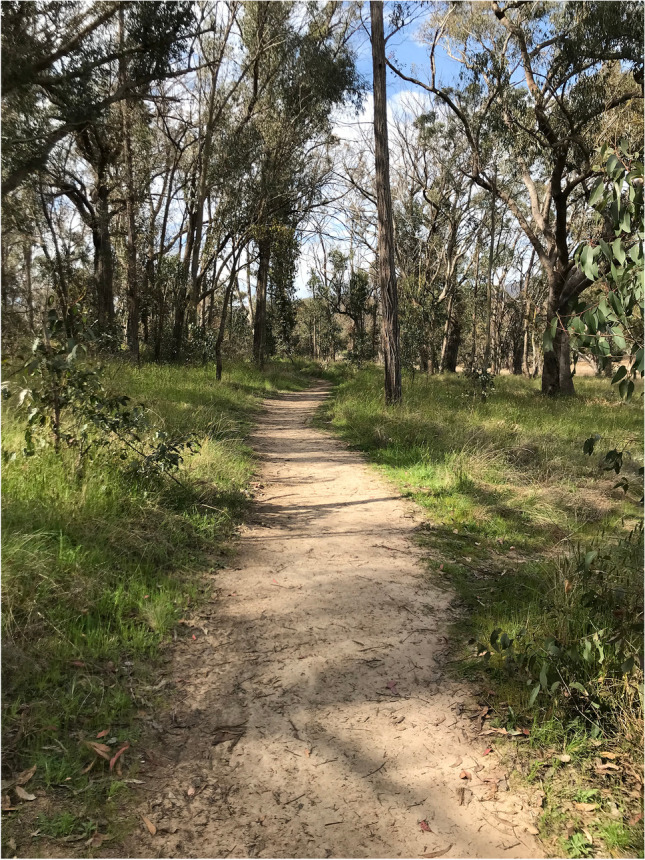
Single-lane walking and running trail at Dumaresq Dam Reserve

The trail around Dumaresq Dam is predominantly a mix of compacted and loose surface single trail with areas of exposed rock (Fig. 2). The surrounding vegetation is open woody grassland, dominated by various *Eucalyptus* species (Debus, Ford et al. 2005). The geology of the area is dominated by Mount Duval adamellite, a granitic geological formation known as the New England Batholith (Wienbelt 1994, Hunter 2015). Armidale has a temperate climate with warm summers and cool winters and a summer dominant rainfall pattern. The mean annual rainfall from 1994 to 2022 was 772 mm (Bureau of Metorology 2022).

### Study design and plot selection

**Fig. 3:**
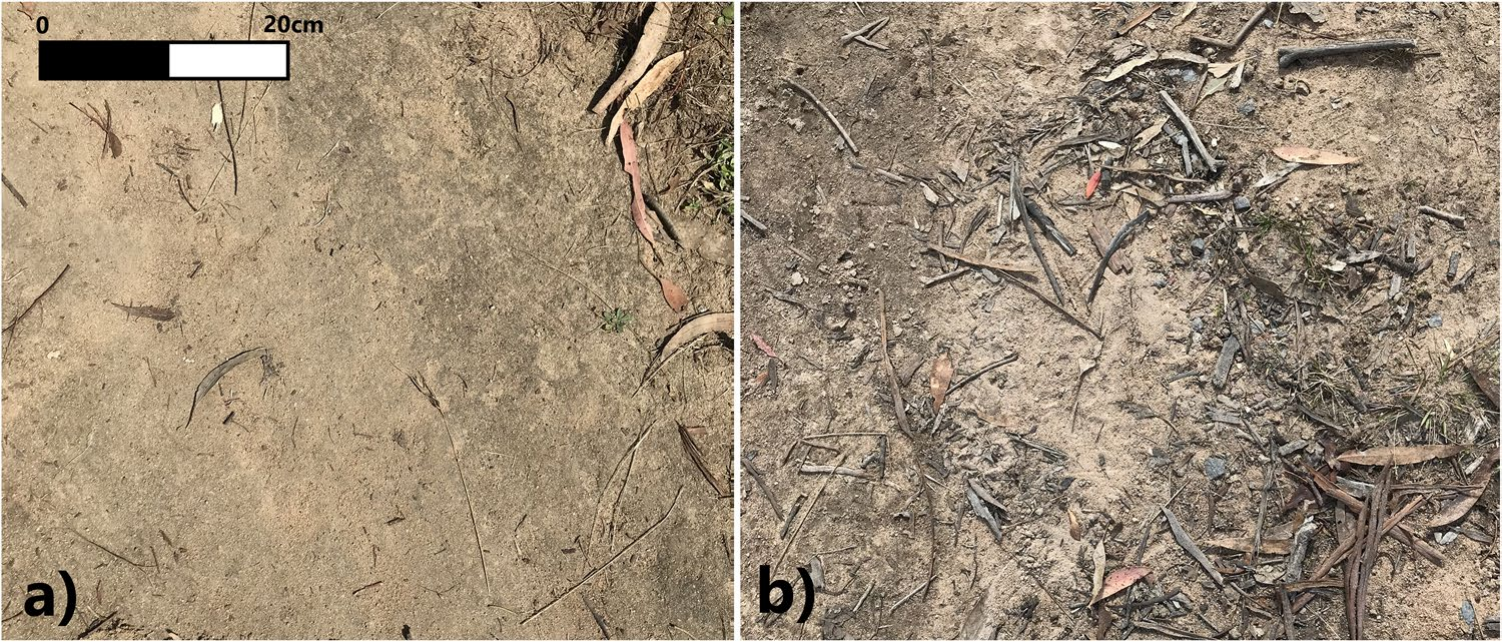
Example of trail surfaces with **a** small and **b** moderate amounts of deposited material

Six different trail surfaces were investigated. These were categorised as small amount of deposited material (loose organic and inorganic material) and no vegetation on three slopes (0–5°, 5–10°, 10–15°), small amount of deposited material with moderate grass cover on a low slope (0–5°), moderate amount of deposited material and no vegetation on a low slope (0–5°) and moderate deposited material on a low slope (0–5°) with grass (Table 1). Landscape function analysis (LFA—see the “Trail surface characterisation” section) classes for the amounts of deposited material were ascribed based on the depth and percent cover of the overlaying soil and material (Tongway and Hindley 2004). Examples of LFA classes for small and moderate amounts of deposited material are depicted in Fig. 3.

**Table 1:** Types of trail surfaces used to investigate microplastic mobility

Group	Plots	Deposited material^1^		Slope (°)	Vegetation
		Amount	LFA score		
Bare, compacted, low slope	*n* = 5	Small	4	<5°	None
Bare, compacted, medium slope	*n* = 5	Small	4	5–10°	None
Bare, compacted, high slope	*n* = 2	Small	4	10–15°	None
Vegetated, compacted, low slope	*n* = 4	Small	4	<5°	Grass, 15–50% cover
Bare, loose, low slope	*n* = 5	Moderate	2	<5°	None
Vegetated, loose, low slope	*n* = 5	Moderate	2	<5°	Grass, 15–50% cover

^1^Detailed information is available in Table S1 and in Tongway and Hindley 2004

Plots for rain simulation were selected on trail sections where surface characteristics were consistent across a 0.4 × 0.4 m (0.16 m^2^) plot area. Exposed roots and large rocks were avoided as hard objects impeded assembly of the simulator. Trail sections steeper than 10° had dense, woody root systems and only two plots were identified where it was possible to install the rain simulator (Table 1). Likewise, vegetated trail surfaces had thick root systems and only four plots were identified on compacted surfaces that were suitable for rain simulation. Other surfaces had five replicates.

### Trail surface characterisation

Trail surfaces were characterised using a combination of LFA methodology and measurements of additional physical properties. LFA is a rapid soil surface monitoring approach and describes the erosion, movement and retention of resources (i.e. sediment and nutrients) in the landscape based on topographical and soil surface attributes (Tongway and Hindley 2004). Resources are described as moving with runoff through relatively bare areas (‘inter-patches’) until they encounter a ‘patch’ of vegetation, debris, low slope or area of high micro-relief.

Soil surface indicators were assessed for each 0.4 m × 0.4 m plot prior to rainfall simulations. Surface roughness, surface resistance to disturbance, crust brokenness, slaking over 10 s, and erosion type and severity were assessed based on the scoring system of LFA (Table S1). Soil texture was determined by hand using the ribbon method and scored as per the corresponding LFA classification. In addition, percent herbaceous and litter cover were estimated visually. It was not possible to assemble the rain simulator on trail surfaces with large root systems or rocks and subsequently the percent root and >2 cm rock cover was negligible for all plots. The slope of the plot was calculated based on the total fall over 0.4 m. The average soil compaction was measured using a handheld penetrometer (ST207 Penetrometer, Zoli Maurizio, Alfonsine, Italy), which was inserted into the trail surface five times at random. The penetrometer readings were taken to a depth of 2.2 cm.

Duplicate soil samples (0–4 cm depth) were collected adjacent to each rainfall simulation plot using a cylindrical steel core cutter (diameter 7 cm) to measure soil moisture and bulk density. Soil moisture was calculated by measuring the dry weight of the soil after drying in an oven at 105°C for 24 h (Black 1965). The bulk density was calculated based on the dry weight of the soil and the volume of the steel core cutter.

The three LFA indicators of soil surface function—stability, infiltration and nutrient cycling indices—were calculated using the supplied LFA spreadsheet (Tongway and Hindley 2004). Plots were identified as patches or inter-patches (resource accruing or shedding areas respectively) based on the relative amounts of deposited material and grass cover, and key trail surface properties are described in Table 2.

**Table 2:** Soil surface characteristics for different trail surfaces. Stability (%) and infiltration (%) indices were calculated using landscape function analysis. Values are means ± SE. Different lower-case letters denote statistically different groups.

Group	Stability (%)	Infiltration (%)	Slope (°)	Compaction (kg/cm^2^)	Bulk density (g/cm^3^)	Soil moisture (%)	Herbaceous cover (%)	Litter cover (%)	Roughness (unitless)	Resistance to disturb. (unitless)	Slake test (unitless)	Texture (unitless)
Bare, compacted, low slope	52.0 ± 1.2b	21.5 ± 0.5a	3.8 ± 0.5a	5.7 ± 0.3a	1.4 ± 0.0a	8.6 ± 1.7ab	1 ± 0a	4 ± 1a	1.0 ± 0.0a	3.8 ± 0.2b	3.8 ± 0.2b	3.8 ± 0.2bc
Bare, compacted, medium slope	47.5 ± 1.4b	27.1 ± 4.8ab	7.5 ± 0.3b	5.9 ± 0.1a	1.3 ± 0.1a	5.8 ± 1.6a	1 ± 0a	13 ± 7ab	1.0 ± 0.0a	4.0 ± 0.0b	3.0 ± 0.0b	4.0 ± 0.0c
Bare, compacted, high slope	48.8 ± 1.3b	22.2 ± 2.9ab	11.5 ± 0.3b	5.1 ± 0.9a	1.4 ± 0.0a	15.4 ± 2.0b	1 ± 1a	2 ± 0a	1.0 ± 0.0a	3.5 ± 0.5b	3.5 ± 0.5b	3.0 ± 0.0ab
Vegetated, compacted, low slope	51.9 ± 4.7b	37.1 ± 6.8bc	3.1 ± 0.6a	5.6 ± 0.4a	1.5 ± 0.1a	13.0 ± 0.8b	24 ± 6b	13 ± 6ab	3.0 ± 0.0c	3.8 ± 0.3b	3.0 ± 0.7b	2.5 ± 0.3a
Bare, loose, low slope	33.1 ± 1.6a	59.6 ± 7.1c	4.0 ± 0.5a	5.5 ± 0.5a	1.3 ± 0.1a	7.3 ± 0.8ab	0 ± 0a	30 ± 9b	1.8 ± 0.2b	1.0 ± 0.0a	1.0 ± 0.0a	3.8 ± 0.2bc
Vegetated, loose, low slope	29.1 ± 0.6a	40.4 ± 0.0bc	4.8 ± 0.2a	4.1 ± 0.6a	1.5 ± 0.1a	11.9 ± 1.1b	36 ± 4b	4 ± 1a	3.0 ± 0.0c	1.0 ± 0.0a	1.0 ± 0.0a	4.0 ± 0.0c

### Trail spiking

Microplastics shed by footwear and clothing are expected to account for the majority of MP on trail surfaces. Microplastics acting as a proxy for shoe abrasion were prepared from a pink shoe outsole made of blown rubber as per Forster et al. (2022). The density of the rubber outsole material (σ = 1.1 g cm^−3^) was determined based on the mass and volume of three samples (with the volume determined using the water displacement method (Flowers, Theopold et al. 2019)). Rubber MPs were used to assess MP mobility as shoe abrasion may be a significant contaminant in areas with high foot traffic (Forster, Tighe et al. 2020) and leachates have been shown to have phytotoxic effects (Lee, Kim et al. 2022). Coloured rubber MPs were selected for the study as they were easier to detect in an organo-mineral matrix than darker particles, and preliminary analysis indicated pink MPs were not already present on the trail surfaces of this study. Particles were linear to globular in shape, and all were light pink. The abraded MPs were measured using a Nikon SMZ745T stereomicroscope (Nikon Corporation, Tokyo, Japan) with Capture 2.1 software (Tucsen Photonics Co., Fuzhou, Fujian, China). Particles >100 μm were used for the study and the longest dimension ranged from 100 to 940 μm. Twenty-six samples were used for the spiking study, consisting of an average of 99 ± 2 SE MPs (each sample individually counted and checked to ensure background contamination was not present). Immediately prior to beginning each rain simulation, the 0.4 m × 0.4 m plots were divided into four quadrats and 25 ± 0.5 particles were dispersed onto the soil surface of each quadrat using a small brush. Rain simulation was not conducted on additional trail surfaces due to the start of above average and ongoing rainfall in the study area.

### Rain simulation

A custom built, battery powered rainfall simulator was used to apply rainwater to the 0.4 × 0.4 m plots, with two to five replicate plots conducted on each trail surface type (Table 1). The simulator comprised a metal frame with a 4-cm grid of hosing that delivered raindrops of 4.8-mm average diameter from a height of approximately 45 cm and with a spatial coefficient of variation of 10%. We used a high rainfall intensity to simulate storm events of 100 mm h^−1^. Field rainfall simulation experiments lasted 15 min. The Australian Bureau of Meteorology Design Rainfall Data System shows the simulated rainfall parameters match a 10% annual exceedance probability (AEP) storm for the area (Bureau of Meterology 2022). Twenty-six rainfall simulations were undertaken in late autumn 2021 when topsoil (0–4 cm) moisture conditions were 10 ± 1% (w/w). All rain simulation tests were at least 3 days after moderate rainfall.

The rainfall simulation plots were bordered by a thin metal sheet, and a stainless-steel flume was inserted on the downslope side of the plot to collect runoff. Plots were placed in the centre of the trail. Runoff was timed and collected in transparent polypropylene vials approximately every 50 mL, with the exact volume later determined accurately via weight. Runoff values were plotted over time to generate runoff hydrographs (Figures S1 and S2), and time to runoff (min) was defined as the time when the first drop of runoff was collected in the polypropylene vial. Rainwater was used from a rainwater storage tank at the University of New England, Armidale, NSW. Water samples were taken every time the simulator water tank was re-filled to monitor MP contamination and each batch was tested for electrical conductivity. The rainwater used in the experiments had an average electrical conductivity of 0.41 dS m^−1^, which met the electrical conductivity standard suggested by Borselli et al. (2001). Background MP contamination of rainwater water was determined by collecting five 50-mL samples of rainwater exiting the rain simulator hosing.

### Microplastic extraction and sediment measurement

The control and runoff samples were suction filtered using pre-weighed 11-μm cellulose filter paper and the sides of the polypropylene vials were rinsed using deionised water. The wet filter paper and filtrate (consisting mainly of sediment, with a small amount of organic matter and MPs) were then transferred into 150-mm polystyrene petri dishes and left at room temperature until completely dry. The lid was placed over the petri dish paper with a 5-mm air gap to allow evaporation while preventing microplastic contamination. Sediment production (comprising mainly of inorganic surface material but with some trace organic material) was measured by weighing the dry filter paper and filtrate, and deducting the weight of the dry filter paper. A white cotton laboratory coat and blue nitrile gloves were worn during filtering to reduce contamination.

### Microplastic detection and characterisation

Spiked and in situ MPs (>100 μm) in all filtered runoff samples were visualised and counted using a Leica EZ4 microscope at ×35 magnification (Forster, Tighe et al. 2022). A MP with known dimensions (100-μm diameter) was used as a visual reference; however, the exact size of individual MPs could not be determined as the microscope was not equipped with a camera. The pink MPs used to spike the trail surfaces were visually distinctive and did not resemble in situ MPs detected in control samples from the same trail. Particles were identified as in situ MPs based on physical properties (colour, shape and sheen) that distinguished them from organic matter and minerals as per Forster et al. (2022). In a previous study by the same laboratory, quantitative recovery of rubber MPs >100 μm was achieved using the same extraction and identification techniques (i.e. suction filtration and microscopy) (Forster, Tighe et al. 2022). The wet surface precluded absolute checks of MP retention on the trail surface following rain simulation; however, given the previous quantitative recoveries, all spiked MPs not detected in the runoff were assumed to have been retained on the trail surface. Microplastic counts for each vial of runoff were corrected by deducting the mean number of MPs in the method blanks (five 50-mL samples of rainwater exiting the rainfall simulator hosing). The time interval to when the first MPs were detected in the trail runoff was measured from the beginning of rainfall. The runoff volume before the first MPs were detected in the trail runoff was measured from the start of runoff.

A subset of MPs was measured and characterised using an Agilent 8700 Laser Direct Infrared Imaging (LDIR) system with Clarity v1.5.0 software (Agilent, Melbourne, Australia). In order to assess the range of MP types present on trail surfaces and determine potential sources, runoff samples from a vegetated loose surface and a bare compacted surface were analysed. Trail runoff particles were transferred directly from the filter paper into a glass scintillation vial using a small brush, then transferred to slides for analysis. Microplastic recovery for different sized particles has not been established for this method of particle transfer and MP counts are considered qualitative. Laser direct infrared imaging is a laser-based imaging and spectroscopy technique that utilises a quantum cascade laser with single-point mercury cadmium telluride detector. Spectra were generated for individual particles with diameters from 20 to 500 μm in the mid-infrared range (975 to 1800 cm^−1^) and compared against the software reference library to identify MPs. Larger diameter MPs cannot currently be analysed using automated LDIR due to size constraints; however, MP length may exceed 500 μm. The longest dimension was reported. Polymer types were determined using a confidence threshold of 75%.

### Data analysis

All data was analysed using R Studio version 2021.9.2.382 (RStudio Team 2022) and R version 4.1.2 (R Core Team 2021). Comparisons of trail surface characteristics, hydrological and erosional responses, and MPs in the trail runoff (identified using microscopy) were analysed using a general linear model specifying a Gaussian, quasigamma or quasipoisson distribution as appropriate. Multiple comparisons were assessed using multcomp (Hothorn, Bretz et al. 2008) and multcompView (Graves et al. 2019) libraries. The association of slope with other trail characteristics and MP mobility with hydrological responses were assessed using Spearman’s correlation.

Conditional inference tree analysis was used to identify the hierarchical importance of trail surface characteristics in explaining variation in MP abundance in the trail runoff. Conditional inference tree analysis is a recursive partitioning method which provides unbiased testing of categorical and continuous variables. During analysis, the variable with maximum explanatory power for MP mobility was identified. The threshold value that split the samples into two groups was identified, and the statistical significance of the association was determined. This process was repeated across all remaining variables until a set of groupings were identified and no further variation could be explained. Comparisons of secondary trail surface characteristics in each node were evaluated using a general linear model specifying a quasigamma or quasipoisson distribution, followed by multiple comparisons. Conditional inference tree analysis was undertaken using the cTREE function in the party library (Hothorn, Hornik et al. 2006). A *p* value of 0.05 or less was taken as indicating statistical significance.

## Results

### Soil surface indicators

The trail sections comprised compacted soils, with measurements exceeding the penetrometer maximum of 6 kg cm^−2^ on 65% of the trail surfaces. There were no statistically significant differences in compaction based on the penetrometer measurements (Table 2, *F*_5,20_ = 1.97, *p* = 0.12). However, semi-quantitative LFA assessments determined soil compaction differed between the compacted trail surfaces with minimal deposited material, and the loose trail surface with moderate amounts of deposited material (*F*_5,20_ = 149.79, *p* < 0.01). The compacted trail surfaces were hard or moderately hard to disturb using a metal implement (resistance to disturbance LFA scores 3–4, Table 2), whereas the thin surface component of the loose trail was classified as loose sand that could be easily penetrated (LFA score of 1).

Likewise, soil slaking (*F*_5,20_ = 27.33, *p* < 0.01) and stability indices (*F*_5,20_ = 30.09, *p* < 0.01) differed on the compacted and loose trail surfaces. Soil aggregates from the compacted surfaces were moderately to very stable when immersed in water for 10 s (LFA slaking scores 3–4), whereas the loose sand dispersed rapidly (LFA slaking score of 1). Compacted trail surfaces had higher LFA stability indices, ranging from 40.0 to 62.5%. The loose surfaces were less stable, ranging from 28.1 to 37.5%.

Infiltration indices significantly differed for the trail surfaces (*F*_5,20_ = 10.17, *p* < 0.01), with a trend towards reduced infiltration on the bare compacted trail surfaces, varying from 19.1 to 40.7% (including all slopes). The mean infiltration index was higher on the vegetated compacted surfaces, but showed high variation (24.6–51.9%). Infiltration was highest on the bare loose surfaces (31.6–70.4%).

Roughness indices were statistically different on the trail surfaces (*F*_5,20_ = 99.43, *p* < 0.01). The bare compacted surfaces (grouped together) had the smoothest surfaces (LFA score of 1), followed by the bare loose surface (LFA scores 1–2). The vegetated surfaces (grouped together) had the highest roughness index (LFA score of 3).

Slope was not strongly associated with any change in surface properties for bare, compacted trail surfaces across the range 2.1–11.7°, with no correlation between increasing slope and stability, infiltration, compaction, bulk density, roughness, litter, or soil texture (ρ value less than 0.4 in all cases, *p* > 0.5). There were no significant differences in bulk density on the trail surfaces (*F*_5,20_ = 1.03, *p* = 0.43). Although there was significant variation in soil moisture and texture, these did not appear consistent with deposited material, vegetation, or slope.

### Hydrological and erosional responses

There was a trend towards delayed runoff on the bare loose soil surfaces (14.8 ± 7.9 min), but there was high variation in results (Table 3). Time to runoff was shortest on the bare compacted trail surface with a medium slope (2.1 ± 0.2 min). The vegetated surfaces each had similar results, with delays of 5.8 ± 0.9 min for the compacted surfaces and 5.0 ± 1.8 min for the loose surfaces.

**Table 3:** Hydrological and erosional responses for different trail surfaces after 15 min of rain simulation. Values are means ± SE. Different lower-case letters denote statistically different groups

Group	Time to runoff (mins)	Total runoff (mL)	Runoff as proportion of total rainfall (%)	Sediment (g)
Bare, compacted, low slope	5.3 ± 1.1ab	481 ± 77b	9 ± 1ab	0.29 ± 0.08a
Bare, compacted, medium slope	2.1 ± 0.2a	486 ± 84b	11 ± 2b	0.62 ± 0.15a
Bare, compacted, high slope	3.3 ± 0.1ab	413 ± 97ab	8 ± 2ab	0.35 ± 0.02a
Vegetated, compacted, low slope	5.8 ± 0.9ab	377 ± 77ab	7 ± 2ab	0.20 ± 0.07a
Bare, loose, low slope	14.8 ± 7.9b	331 ± 35ab	5 ± 1a	0.26 ± 0.06a
Vegetated, loose, low slope	5.0 ± 1.8ab	253 ± 24a	5 ± 1a	0.21 ± 0.09a

Runoff volume varied on the trail sections (*F*_5,20_ = 2.75, *p* = 0.05); however, the trail surfaces did not group separately according to vegetation or the amount of deposited material. Although there were no statistically significant differences, there was a weak trend towards reduced runoff volumes on loose and vegetated trail surfaces, with the vegetated loose trail surface generating the least runoff (253 ± 24 mL). Higher runoff volumes were produced on the bare compacted trail surfaces; however, steeper slopes were not associated with increased runoff (481 ± 77 mL on low slope, 486 ± 84 mL on medium slope and 413 ± 97 mL on high slope surfaces).

Total sediment production did not differ on the various trail surfaces (*F*_5,20_ = 2.10, *p* = 0.11). The bare compacted trail surface at a medium slope had the widest range in results and highest upper value, ranging from 0.34 to 1.18 g. There was high variation in sediment production on all trail surfaces, with 0.03–0.32 g produced on vegetated compacted surfaces, 0.06–0.41 g on bare loose surfaces and 0.02–0.44 g on vegetated loose surfaces.

### Microplastics in trail surface runoff

#### Spiked microplastics

The spiked rubber MPs were relatively immobile on the trail surfaces, with 0–15% detected in the runoff after 15 min of simulated rainfall (Table 4). Microplastic mobility differed across the various trail surfaces (*F*_5,20_ = 3.65, *p* < 0.05) and overall, there was a positive relationship between total runoff volume and the percent of spiked rubber MPs detected in runoff (ρ = 0.75, *p* < 0.01).

**Table 4:** Total in situ and spiked microplastics (MP) (>100 μm) in runoff for different trail surfaces after 15 minutes of rain simulation, and total rainfall time and runoff volume before microplastics were detected. Values are means ± SE. Different lowercase letters denote statistically different groups

Group	Spiked MP (%)	Time to first spiked MP (min)	Runoff volume to first spiked MP (mL)	In situ MP (counts)	Time to first in situ MP (mins)	Runoff volume to first in situ MP (mL)	In situ MP per 100 mL
Bare, compacted, low slope	10.9 ± 2.2c	8.8 ± 1.2ab	59 ± 10a	24.9 ± 4.6a	7.6 ± 0.9a	49 ± 1a	6.2 ± 1.8ab
Bare, compacted, medium slope	6.3 ± 1.5bc	6.8 ±1.2a	80 ± 21ab	18.1 ± 5.4a	5.9 ± 0.7a	49 ± 1a	3.5 ± 0.4a
Bare, compacted, high slope	9.4 ± 4.9bc	8.6 ± 1.5ab	119 ± 72ab	23.6 ± 1.1a	6.9 ± 0.1ab	47 ± 0a	6.1 ± 1.7ab
Vegetated, compacted, low slope	6.1 ± 2.7bc	9.2 ± 1.1ab	61 ± 11a	35.1 ± 9.0a	8.6 ± 0.6ab	50 ± 1a	9.2 ± 1.6b
Bare, loose, low slope	2.7 ± 1.1ab	23.2 ± 6.4b	146 ± 52ab	15.8 ± 2.7a	20.1 ± 7.1b	59 ± 10a	5.2 ± 1.4ab
Vegetated, loose, low slope	1.4 ± 0.6a	17.4 ± 1.7ab	198 ± 20b	24.2 ± 3.4a	8.5 ± 2.0ab	48 ± 1a	10.0 ± 1.9b

Microplastic mobility tended to be higher on compacted surfaces, but results were highly variable. The bare compacted trail surfaces had the most MPs in the trail runoff, with 10.9 ± 2.2% from the low slope, 6.3 ± 1.5% from the medium slope and 9.4 ± 4.9% from the high slope. In comparison, the trail runoff contained 2.7 ± 1.1% from the bare loose surfaces and 1.4 ± 0.6% spiked MPs from the vegetated loose surfaces. Microplastic mobility also tended to be lower on vegetated surfaces, with 44–45% fewer MPs in the trail runoff compared to the bare low slope surfaces with equivalent soil compaction. However, values were variable and this trend was not significant. There was no correlation between spiked MPs in the trail runoff and slope (ρ = 0.034, *p* = 0.87).

Rainfall duration (*F*_5,20_ = 7.79, *p* < 0.01) and runoff volume (*F*_5,20_ = 4.02, *p* < 0.05) before the spiked particles were first detected in the trail runoff differed significantly for the trail surfaces, which however did not group according to vegetation or the amount of deposited material (Table 4, Figure S1). There was no association between slope and rainfall duration or runoff volume before the spiked MPs were first detected in the runoff, with delays of 8.8 ± 1.2 min rainfall (59 ± 10 mL runoff) on the low slope, 6.8 ±1.2 min (80 ± 21 mL runoff) on the medium slope and 8.6 ± 1.5 min (119 ± 72 mL runoff) on the high slope. Lateral MP movement tended to be reduced on the loose soil surfaces, with a total of 23.2 ± 6.4 min rainfall (146 ± 52 mL runoff) on the bare loose surface and 17.4 ± 1.7 min rainfall (198 ± 20 mL) on the vegetated loose trail surface to when the first spiked MP was detected in the trail runoff. The average increase in spiked MPs in the trail runoff as a function of time is shown for each trail surface in Figure S3.

#### In situ microplastics

Overall, there were approximately four times the number of in situ MPs compared to spiked particles in samples. There was wide variation in MP counts, with 9 to 55 in situ MPs (>100 μm) detected in the trail runoff from the rain simulation plots (Table 4). A variety of MPs >100μm were detected using microscopy, with 85% being fibres and 15% being irregular-shaped fragments. There was no correlation in the total number of in situ and spiked MPs in the trail runoff (ρ = 0.09, *p* = 0.63).

There was no significant difference in in situ MP counts for the different trail surfaces and variation was high (*F*_5,20_ = 1.68, *p* = 0.19); however, there was a weak trend towards higher MP counts in 15-min runoff from the vegetated surfaces compared to the bare, low slope surfaces. On average, runoff from the vegetated compacted trail surface had 29% more MPs (35.1 ± 9.0 particles) than the bare compacted trail surface (24.9 ± 4.6 particles), and the runoff from the vegetated loose surface (24.2 ± 3.4 particles) had 35% more than the bare loose surface (15.8 ± 2.7 particles) (Table 4). Runoff from the vegetated trail surfaces contained more MPs per runoff volume (*F*_5,20_ = 2.90, *p* <0.05), with 9.2 ± 1.6 MP/100 mL from the compacted surface and 10.0 ± 1.9 MP/100 mL from the loose surface. In comparison, the mean MPs in the runoff varied from 3.5 ± 0.4 MP/100 mL for the bare, compacted, medium slope to 6.2 ± 1.8 MP/100 mL on the bare, compacted, low slope. The number of in situ MPs in the trail runoff was not correlated to slope (ρ = −0.30, *p* = 0.14), with fewer particles in the runoff from the bare compacted medium slope (18.1 ± 5.4) compared to the low and high slope surfaces.

Time to runoff and total rainfall duration before the first in situ MPs appeared in the trail runoff were closely associated (ρ = 0.87, *p* < 0.01) and there were statistically significant differences between the trail surfaces before the first in situ MPs were detected in the runoff (*F*_5,20_ = 4.85, *p* < 0.01) (Table 4, Figure S2). Microplastics were first detected in the trail runoff for the bare compacted medium slope surfaces after 5.9 ± 0.7 min rainfall, followed by the high slope surfaces (6.9 ± 0.1 min) and low slope surfaces (7.6 ± 0.9 min). Microplastics were first detected in the runoff from the vegetated surfaces after similar periods of rainfall (8.6 ± 0.6 min and 8.5 ± 2.0 min respectively for compacted and loose surfaces). The lag period before the first particle was detected in the runoff was longest on the bare, loose trail surfaces (20.1 ± 7.1 min). In situ MPs were detected in the first vial of runoff from 96% of the simulation plots and there was no statistical difference in the runoff volume before the first microplastic particle was detected (*F*_5,20_ = 1.03, *p* = 0.43). The average increase in MPs in the trail runoff as a function of time is shown for each trail surface in Figure S3.

Samples from two surfaces were analysed in order to acquire a preliminary indication of the range of MP types present on trails, including a vegetated loose surface and a low slope bare compacted surface. LDIR identified 106 MPs ranging from 23 to 640 μm in size (Table 5). There were twice the number of MPs in the size fraction 23–100 μm compared to larger MPs (>100 μm). The most abundant MP types >100 μm were polyurethane (43%), followed by polypropylene (17%), and polyethylene terephthalate (11%). The most abundant MPs in the smaller size fraction were polyurethane (32%), polyamide (18%) and polyethylene terephthalate (13%). Comparisons of the number of MPs >100 μm acquired using microscopy and LDIR are shown in Table S2. The number of MPs identified using microscopy (31 MPs) was substantially higher than LDIR (2 MPs) for the vegetated loose surface (31 MPs compared to two). In comparison, slightly more MPs were detected in the runoff from the bare compacted surface using LDIR (33 MPs versus 27 MPs identified using microscopy).

**Table 5:** Types of microplastics (<100 μm and >100 μm) identified in runoff from vegetated, loose, low slope and bare, compacted, low slope trail surfaces. Microplastics were detected using LDIR and the polymer type was identified by matching spectra against a reference library using a 75% confidence threshold

Polymer	Microplastics <100 μm		Microplastics >100 μm	
	Counts	Percent (%)	Counts	Percent (%)
Polyurethane (PU)	23	32%	15	43%
Polypropylene (PP)	6	8%	6	17%
Polyethylene terephthalate (PET)	9	13%	4	11%
Polyvinylchloride (PVC)	1	1%	3	9%
Polyethylene (PE)	2	3%	2	6%
Polystyrene (PS)	6	8%	1	3%
Alkyd varnish	4	6%	1	3%
Rubber	1	1%	1	3%
Polytetrafluoroethylene (PTFE)	0	0%	1	3%
Polycarbonate (PC)	1	1%	1	3%
Polymethylmethacrylate (PMMA)	3	4%	0	0%
Polyamide (PA)	13	18%	0	0%
Silicone	1	1%	0	0%
Ethylene vinyl acetate (EVA)	1	1%	0	0%
**Total**	71	100%	35	100%

### Associations between microplastic mobility and trail surface characteristics

#### Hierarchy of explanatory variables

**Fig. 4:**
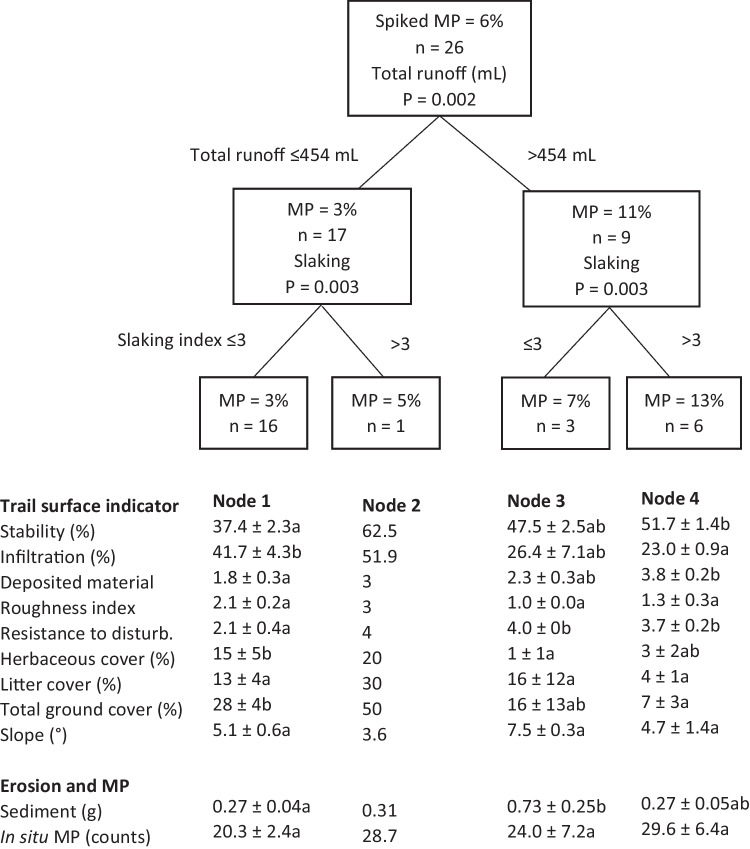
Conditional inference tree for the percent spiked MPs (MP) in trail runoff, showing the significance of explanatory variables at each tree split (with *p*-value), critical variable partitioning into groups and resulting spiked MPs (%) at each node. Soil surface indicators are tabulated below each terminal node. Different lowercase letters denote statistically different groups

The conditional inference tree for the percentage of spiked MPs in trail runoff had four splits and four terminal nodes (terminal nodes = final groupings of data) (Fig. 4). The strongest explanatory variable for lateral movement of the spiked MPs was total runoff volume, with a critical threshold value of 454 mL. Simulation plots producing >454 mL runoff after 15 min rain simulation had an average of 11% of the spiked MPs in the trail runoff, compared to 3% for simulation plots producing ≤454 mL runoff.

Slaking was the next strongest and final explanatory variable in the conditional inference tree analysis. The node 1 rain simulation plots had ≤454 mL runoff and slaking LFA scores ≤3 (unstable soil aggregates that dispersed rapidly in water) and had the fewest MPs (3%) in the trail runoff. Node 2 (containing one simulation plot) had ≤454 mL runoff and a LFA slaking score >3 (soil aggregates were stable in water for at least 10 s) and had 5% spiked MPs in the trail runoff. Node 3 rain simulation plots had >454 mL runoff and LFA slaking scores ≤3 (all were classified as moderately stable) and had an average of 7% spiked MPs in the runoff. Node 4 rain simulation plots had >454 mL runoff and very stable soil aggregates (LFA slaking scores >3) and had 13% MPs in the trail runoff. No tree pattern was detectable for in situ MPs.

#### Secondary trail surface characteristics related to microplastic mobility and retention

Secondary trail surface characteristics related to MP mobility and retention were identified using data for nodes 1, 3 and 4, which had *N* ≥ 3 (Fig. 4). Surface indicators associated with node 1 were related to MP retention on trails, whereas surface indicators associated with node 4 were related to MP mobility. Node 3 was intermediate, sharing characteristics with nodes 1 and 4.

There were statistically significant differences between nodes for soil stability (*F*_2,22_ = 6.25, *p* < 0.01), infiltration (*F*_2,22_ = 6.05, *p* < 0.01), deposited material (*F*_2,22_ = 6.39, *p* < 0.01) and total ground cover (including litter and herbaceous cover) (*F*_2,22_ = 5.30, *p* < 0.05), with nodes 1 and 4 grouping separately.

Increased MP mobility on trail surfaces was associated with increased soil compaction, lower soil permeability and less retained material. Node 4 trail surfaces had the highest soil stability (51.7 ± 1.4%), lowest infiltration (23.0 ± 0.9%) and least amount of deposited material (LFA score of 3.8 ± 0.2) and overall ground cover on the trail surface (7 ± 3%). Conversely, node 1 trail surfaces had the lowest soil stability (37.4 ± 2.3%), highest infiltration (41.7 ± 4.3%) and greatest amount of deposited material (LFA score of 1.8 ± 0.3) and ground cover (28 ± 4%). Node 3 trail surfaces were intermediary, with LFA scores of 47.5 ± 2.5% for soil stability, 26.4 ± 7.1% for infiltration, 2.3 ± 0.3 for deposited material and 16 ± 13% total ground cover.

Soil compaction varied between the nodes, with LFA scores for resistance to disturbance grouping separately for nodes 3 and 4 (*χ*^2^ = 14.64, *p* < 0.05). The trail surfaces included in nodes 3 and 4 were classified as hard or moderately hard to mechanically disturb using a metal implement (LFA scores 3–4), whereas the node 1 trail surfaces had an average score of 2.1 ± 0.4 (easily broken).

Reduced MP mobility on trail surfaces was associated with higher topographical variation and vegetation; however, there was not a linear relationship between increased mobility and individual surface indicators. There were significant differences between nodes for roughness (*F*_2,22_ = 4.05, *p* < 0.05) and herbaceous cover (*F*_2,22_ = 3.34, *p* = 0.05), with node 1 and node 3 grouping separately. Node 1 had the most topographical variation, with a roughness score of 2.1 ± 0.2 and 15 ± 5% herbaceous cover. Node 3 had the least, with a roughness score of 1.0 ± 0.0 and 1 ± 1% herbaceous cover. Node 4 was intermediary, with a roughness score of 1.3 ± 0.3 and 3 ± 2% herbaceous cover.

There were no significant differences between nodes for litter cover (*F*_2,22_ = 2.24, *p* = 0.13) and slope (*F*_2,22_ = 4.90, *p* = 0.37).

## Discussion

Outdoor recreation is a major source of MPs on trails in conservation and wilderness areas due to abrasion of footwear and clothing (Forster, Tighe et al. 2020, Forster, Tighe et al. 2022). The majority of in situ MPs >100 μm in the trail runoff in this study were fibres, which can be attributed to synthetic clothing. The chemical composition was determined for a subset of MPs (20–640 μm in length), with the majority comprising polyurethane, polyester and nylon, which can be attributed to shoe outsoles and synthetic clothing. Rubber MPs from shoe abrasion may be underestimated as particles may be as small as 1–5 μm, and characterisation of black particles remains very challenging for microscopy and spectroscopy techniques (Barry and Milburn 2013, Wu, Zhou et al. 2016). Our findings match preliminary research on MP pollution in snow and rivers of wilderness areas with trails, where nylon, polyester and rubber were attributed to clothing, mountain bike tires and shoes (Barrows, Christiansen et al. 2018, Napper, Davies et al. 2020). In the sole study investigating plastic pollution on mountain trails, food packaging, mountaineering equipment and clothing accounted for the majority of larger macroplastics (plastic particles >5 mm) that contained polyethylene, polystyrene, polypropylene, polyester and polyurethane (Parolini, De Felice et al. 2021). A wide range of other polymer types were identified in the trail runoff, indicating that MP pollution from hiking and trail running may have a mix of biological, microbiological, chemical and physical effects on the soil environment (Ingraffia, Amato et al. 2021, Colzi, Renna et al. 2022, Lahive, Cross et al. 2022, Lee, Kim et al. 2022, Sun, Duan et al. 2022). Further research is required to determine the implications of MPs on ecological processes and plant and animal species in conservation and protected areas, particularly polyurethane fragments and polyester and nylon fibres. Microplastics retained on trails may impact the soil environment within a relatively limited area; however, MPs with increased mobility may have widespread impacts.

Research to date has focussed on wind, soil processes and animals as vectors for short- and long-range MP translocation in the terrestrial environment (O'Connor, Pan et al. 2019, Yu, van der Ploeg et al. 2019, Bullard, Ockelford et al. 2021). Systematic analysis of the role of rainfall in the redistribution of MPs—particularly on irregular surfaces—has been minimal (Zhang, Liu et al. 2020, Laermanns, Lehmann et al. 2021, Zhang, Chen et al. 2022). In this study, simulated rainfall on spiked trail surfaces was used to examine the impact of soil surface conditions on lateral movement of MPs during a 10% AEP storm for the area. Retention of spiked MPs was very high across all trail surfaces, matching preliminary research that found 53% of MPs on fine- and coarse-grain surfaces remained completely stationary when exposed to runoff for 50 s (Laermanns, Lehmann et al. 2021). Previous analysis from the same trail detected 333 ± 106 MPs per 40 cm^2^ on bare, compacted surfaces and 277 ± 60 MPs per 40 cm^2^ on bare, loose surfaces (Forster, Tighe et al. 2022). Based on these results, it appears in situ MP retention may be commensurate and as high as 93–94%, indicating MP retention may be widespread on trails during short storm events. The majority of in situ MPs in this study were fibres, highlighting the need for rain simulation studies specifically evaluating the movement of fibres with rainfall. The total number of spiked and in situ MPs in the trail runoff did not correlate, which may be due to differences in mobility of fibres and fragments, or because of variation in the number of in situ MPs on different trail surfaces due to long-term patterns of microplastic deposition and movement.

Microplastics are a heterogeneous class of contaminants and may exhibit very wide variation in properties that affect mobility, such as buoyancy. The density of commonly found MPs can range from 0.8 to 0.9 g cm^−3^ for polyethylene and polypropylene, and up to 1.1–1.6 g cm^−3^ for polyamide, polyester and polyvinyl chloride (Chubarenko, Bagaev et al. 2016). The density of rubber and polyurethane may vary from 0.6 to 1.2 g cm^−3^, being impacted significantly by the presence of additives, fillers and micro-voids, as well as crosslinking density (Giese and Brown 1991, Ames 2004). In the environment, MP density may further differ depending on the degree of weathering, biofilm formation and mineral aggregation (Kowalski, Reichardt et al. 2016, Hoellein, Shogren et al. 2019). In combination with trail surface characteristics and low runoff velocity, the high rates of particle retention on the trail surfaces of this study are most likely in part due to the MP density exceeding that of rainwater, as the MPs used for spiking the trail surfaces were produced from rubber material with a density of 1.1 g cm^−3^. Likewise, the polyester and polyamide MPs detected in the trail runoff are likely to be heavier than water. MPs such as low-density polyurethane may be more buoyant and mobile in rainwater runoff, potentially travelling further from trails into protected areas.

For MPs >100 μm that did move during rain simulation, there was a positive relationship between increased runoff volume and lateral MP mobility. Soil compaction has previously been identified as a major factor influencing runoff rates as trail compaction can reduce the relative proportion of macropores (particularly coarse macropores >110 mm), causing reductions in effective and preferential porosity and saturated hydraulic conductivity (Sutherland, Bussen et al. 2001, Cole 2004, Marion, Leung et al. 2016). Compaction was not an explanatory variable in this study for reduced soil permeability and increased trail runoff as a high proportion of measurements exceeded the penetrometer maximum. However, LFA scores for slaking, deposited material and infiltration effectively categorised differences in soil permeability on the trail surfaces. The compacted trail surfaces in this study comprised soils that were stable and did not disperse when exposed to water. There was minimal deposited material on the surface of the trail, limiting infiltration capacity and encouraging runoff production. For the compacted soils in this study, low rates of slaking may reflect reduced macroporosity and the inability of water to infiltrate soil fragments during the relatively short test period. In contrast, trail surfaces with increased MP retention had a layer of loose sand that slumped rapidly when exposed to water and provided increased water infiltration. The loose sand also reduced raindrop impact and the kinetic energy available for lateral MP movement (Hillel 2004).

Surface roughness is known to influence hydrological and erosional responses as micro-topographical variation, and vegetation can physically trap sediment particles and impact runoff velocity (Tongway and Hindley 2004). Previous studies have found that leaf litter also mitigates raindrop impact, reduces particle detachment, increases infiltration and reduces runoff velocity (Danacova, Valent et al. 2017, Du, Niu et al. 2019, Jourgholami, Sohrabi et al. 2022). Likewise, higher rainfall and runoff velocity has been found to increase MP mobility in laboratory conditions and urban environments (de Jesus Piñon-Colin, Rodriguez-Jimenez et al. 2020, Laermanns, Lehmann et al. 2021). Fewer spiked MPs were present in runoff from trail surfaces with vegetation, litter and variable micro-relief, suggesting ground cover obstructed MP movement and/or reduced runoff velocity. In comparison, more in situ MPs were detected in runoff from the same simulation plots, suggesting more MPs had been retained on the trail surface during light to moderate rainfall, but were dislodged when the runoff velocity reached a critical threshold. Runoff rates have been observed to decrease off-trail (Wallin and Harden 1996), suggesting vegetation immediately adjacent to trails may be preferentially enriched with MPs. However, the relative number of MPs on different surfaces may also be influenced by the presence of herbaceous, litter and canopy cover, which may limit thermal and photo-oxidative exposure, thereby slowing degradation and fragmentation of larger MPs.

Our results indicate that MPs are relatively immobile and MPs from recreation may predominantly be found on and adjacent to areas with foot traffic. Key surface characteristics may be used to identify trail sections that are more and less likely to retain MPs (Table 6). Potentially, these surface indicators may be used as a rapid assessment tool to identify likely MP accrual zones in protected areas. There has been very limited research on the spatial distribution of MPs on trail surfaces to validate our findings; however, previous research found approximately four times more MPs (mainly microfibres) on bare, loose surfaces on a walking and running trail (Forster, Tighe et al. 2022), indicating there was increased MP retention on a trail surface with retained alluvium and low resistance to disturbance. Seasonal and climactic conditions leading to storm events and high rainfall may further impact the long-term distribution of MPs in the environment. Trail design, maintenance and visitor numbers may also influence MP distribution as trail compaction, soil texture, trail incision and profile, and slope influence runoff rates and direction of runoff (Liddle 1997, Cole 2004). Overall resource retention on trails may influence the severity of MP impacts on the environment as there may be increased quantities of MPs in accrual areas and greater potential for long-term exposure. However, subsequent deposition and retention of litter and alluvium above MPs may reduce thermal- and photo-oxidative degradation, resulting in reduced rates of leaching compared to weathered particles. Microplastic weathering may also be reduced in forested areas due to shading by surrounding vegetation.

**Table 6:** Trail properties related to microplastic mobility and retention

Soil surface property	Increased microplastic mobility	Increased microplastic retention
Vegetation	No vegetation	Perennial vegetation present
Slake test	Minimal aggregate dispersion	Soil fragments slump within 10 s
Deposited materials	Small amount of deposited material; subsoil or crust is exposed	Layer of retained alluvium
Roughness	Shallow depressions, smooth surface	Roughness due to variation in micro-relief, roots, vegetation, rocks or litter
Resistance to disturbance	High resistance to penetration, hard crust	Soil is easily broken and disturbed, loose sandy surface

For the first time, this study has evaluated the impact of trail surface characteristics on MP movement with rainfall. Extensive and ongoing research is required however to understand transport mechanisms for MPs on soil surfaces, including the relative amounts, types and sizes that are preferentially transported by animals, wind and rainfall. Due to the challenges of using the rain simulator on soils with roots, rocks and steep slopes, this study evaluated the mobility of rubber MPs on a relatively small subset of trail surface types. It is critical to investigate MP mobility on a wider range of surfaces, including gravel, boardwalks and different soil textures and steeper gradients. It is also necessary to examine mobility for various types of MPs—particularly fibres and aged MPs—under different rainfall intensities. In addition, the average soil moisture was low (<10%) when the simulation tests were completed, and trail runoff is likely to differ under saturated conditions and with prolonged rainfall. Furthermore, the horizontal and vertical spatial distribution of MPs on and beside trails should be investigated to validate models of MP transport on irregular soil surfaces. Microplastic deposition rates may vary on under different conditions and it is critical MP shedding is quantified for various types of clothing and footwear, recreation (e.g. hiking vs trail running) and trail surfaces. Research on MP transport faces ongoing challenges as detection, visualisation and quantification are confounded for MPs <100 μm; however, the abundance of smaller MPs identified using LDIR underscores the importance of developing cost-effective identification techniques to expedite research. It is also critical to assess MP recovery for LDIR for a range of size fractions and sample handling techniques. Counts for MPs >100 μm differed for microscopy and LDIR in the samples analysed using both techniques, which may be due to differences in the size of MPs or limitations in the accuracy of microscopy for identification of smaller MPs.

## Conclusion

This study indicates microplastic mobility is relatively low on many recreational trail surfaces during short storm events. Microplastic mobility is predominantly associated with trail surface conditions that facilitate soil compaction and increased runoff velocity, but MPs may accumulate in areas where there is vegetation, topographical variation and/or loose alluvium that reduces runoff rates or physically obstructs microplastic particles. There is potential for a subset of soil surface indicators to be used as a rapid assessment tool to assist land managers in identifying potential accrual zones in ecologically significant areas, enabling decision-making regarding numbers of visitors and trail maintenance. Further research is needed however to assess MP mobility for a broader range of MP physicochemical properties and trail surfaces.

Microplastic impacts on the soil environment may be greater in areas characterised by high retention rates, where there is likely to be higher concentrations of MPs and longer exposure periods. In these areas, MPs may have long-term implications on soil functioning, chemistry, microbiology, organisms and vegetation. However, shading by vegetation and deposition of litter and alluvium may alter weathering characteristics of retained MPs, potentially impacting rates of leaching.

## Supplementary information

ESM 1

## Data Availability

Not applicable.
