# Supplementary material for: Microplastic surface retention and mobility on hiking trails
Source: Environ Sci Pollut Res Int. 2023 Jan 31;30(16):46368–82. doi: 10.1007/s11356-023-25635-z (PMC10097793; doi:10.1007/s11356-023-25635-z)
Supplement: Supplementary file 1 — ESM 1 [file 11356_2023_25635_MOESM1_ESM.pdf]

Supplementary Information

Microplastic surface retention and mobility on hiking trails

Environmental Science and Pollution Research

Nicola Ann Forster[a^], Susan Caroline Wilson[a], Matthew Kevin Tighe[a]

[a] School of Environmental and Rural Science, University of New England, Armidale, Australia

[^] Corresponding author email address:
nforste2@myune.edu.au or nicola.a.forster@outlook.com

Table S1: Description of LFA criteria for rapid assessment of soil surface indicators

Soil Surface Indicator	LFA Scoring System
Deposited Materials<br>(1 – 4)	1 = Greater than 50% cover; several cm deep<br>2 = 20 to 50% cover; moderate amount of material available<br>3 = 5% to 20% cover; slight amount of material available<br>4 = 0-5% cover; none or small amount of material available
Surface Roughness<br>(1 – 5)	1 = <3 mm relief in soil surface<br>2 = Shallow depressions 3-8 mm relief<br>3 = Deeper depressions 8-25 mm, dense tussock grasslands<br>4 = Deep depressions that have a visible base<br>5 = Very deep depressions or cracks >100mm
Surface Resistance to Disturbance<br>(1 – 5)	1 = Loose sandy surface<br>2 = Easily broken<br>3 = Moderately hard<br>4 = Crust is very hard and brittle<br>5 = Non-brittle
Slaking<br>(1 – 4)	1 = Very unstable. Fragment commences slumping in less than 5 sec. Very fine air bubbles may emerge<br>2 = Unstable. Fragment substantially slumps in 5-10 sec. Thin surface crust remains. > 50 % slumps<br>3 = Moderately stable. Surface crust intact. Slumping of sub crust but <50%<br>4 = Very stable. Whole fragment intact. Large air bubbles may emerge
Erosion Type and Severity<br>(1 – 4)	Erosion Type: Sheeting = E; Pedestal =P; Terracette =T; Rill =R; Scalding = S<br>1 = Severe<br>2 = Moderate<br>3 = Slight<br>4 = Insignificant
Crust Brokenness<br>(1 – 4)	0 = No crust present<br>1 = Crust present but extensively broken<br>2 = Crust present but moderately broken<br>3 = Crust present but slightly broken<br>4 = Crust present but intact, smooth
Texture<br>(1 – 4)	1 = silty clay to heavy clay<br>2 = sandy clay loam to sandy clay<br>3 = Sandy loam to silt loam<br>4 = sandy to clayey sand

Table S2: Microplastic counts for subset of MPs detected using both microscopy and LDIR.

	Microscope	LDIR	
Trail surface	MPs >100 µm	<100 µm	>100 µm
Vegetated, loose, low slope	31	15	2
Bare, compacted, low slope	27	56	33

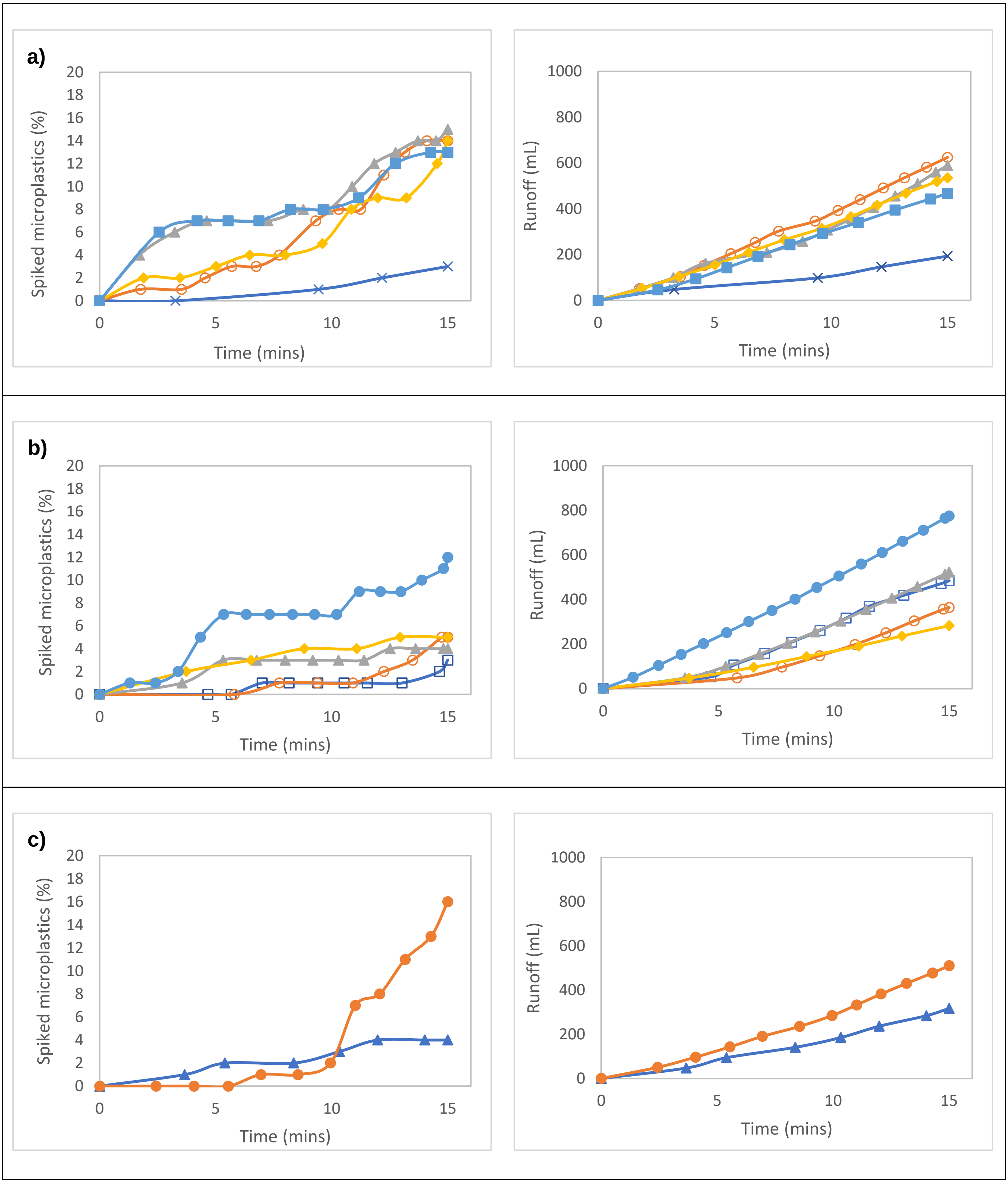

Figure S1a-c: Cumulative spiked microplastics and runoff from trail surfaces, including bare, compacted surface on three slopes; a) 0-5°, b) 5-10°, c) 10-15°). Individual lines represent rain simulation on the replicate plots.

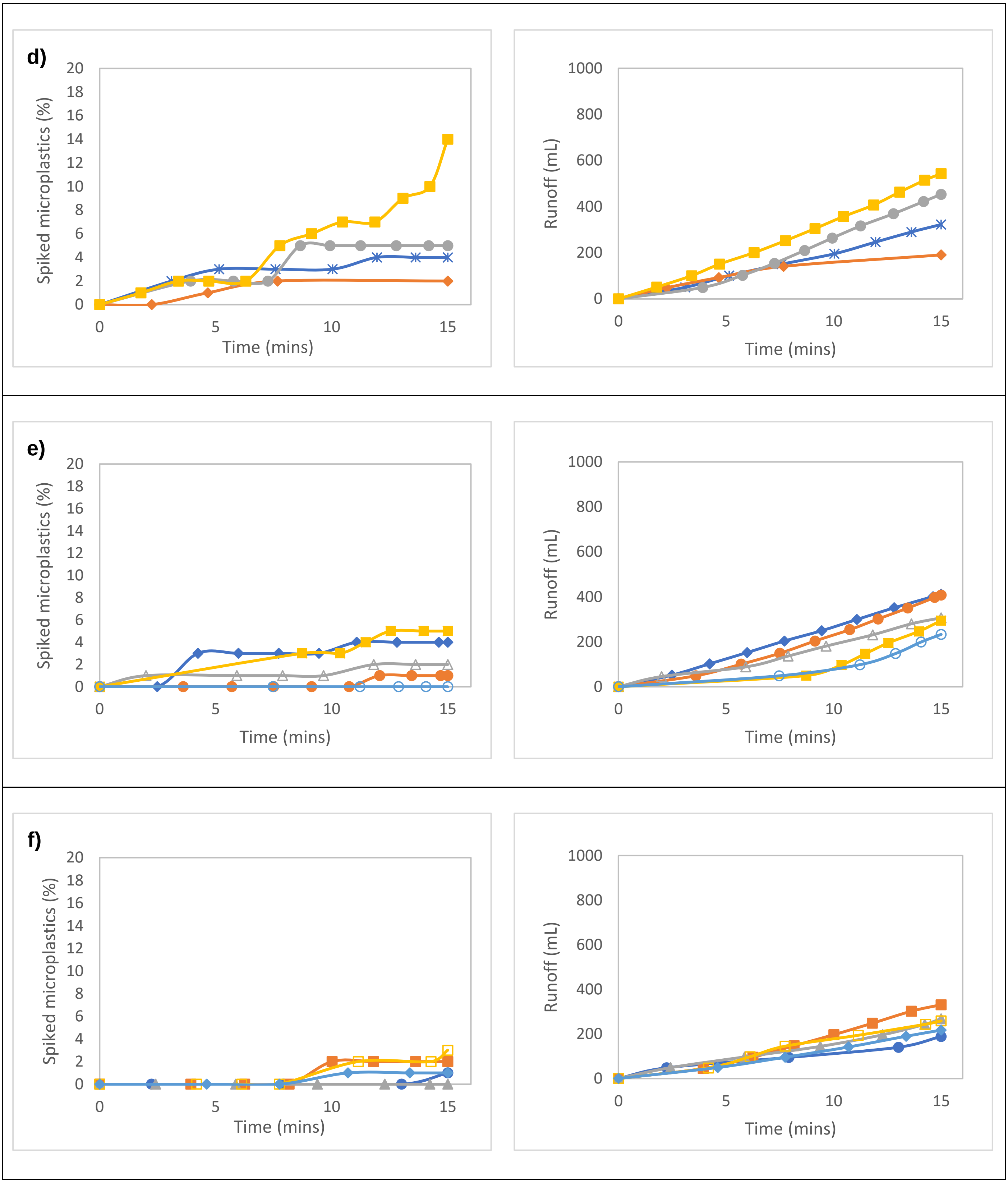

Figure S1d-f: Cumulative spiked microplastics and runoff from trail surfaces, including d) vegetated, compacted surface on a low slope (0-5°), e) bare, loose surface on a low slope (0-5°), and f) vegetated, loose surface on a low slope (0-5°). Individual lines represent rain simulation on the replicate plots.

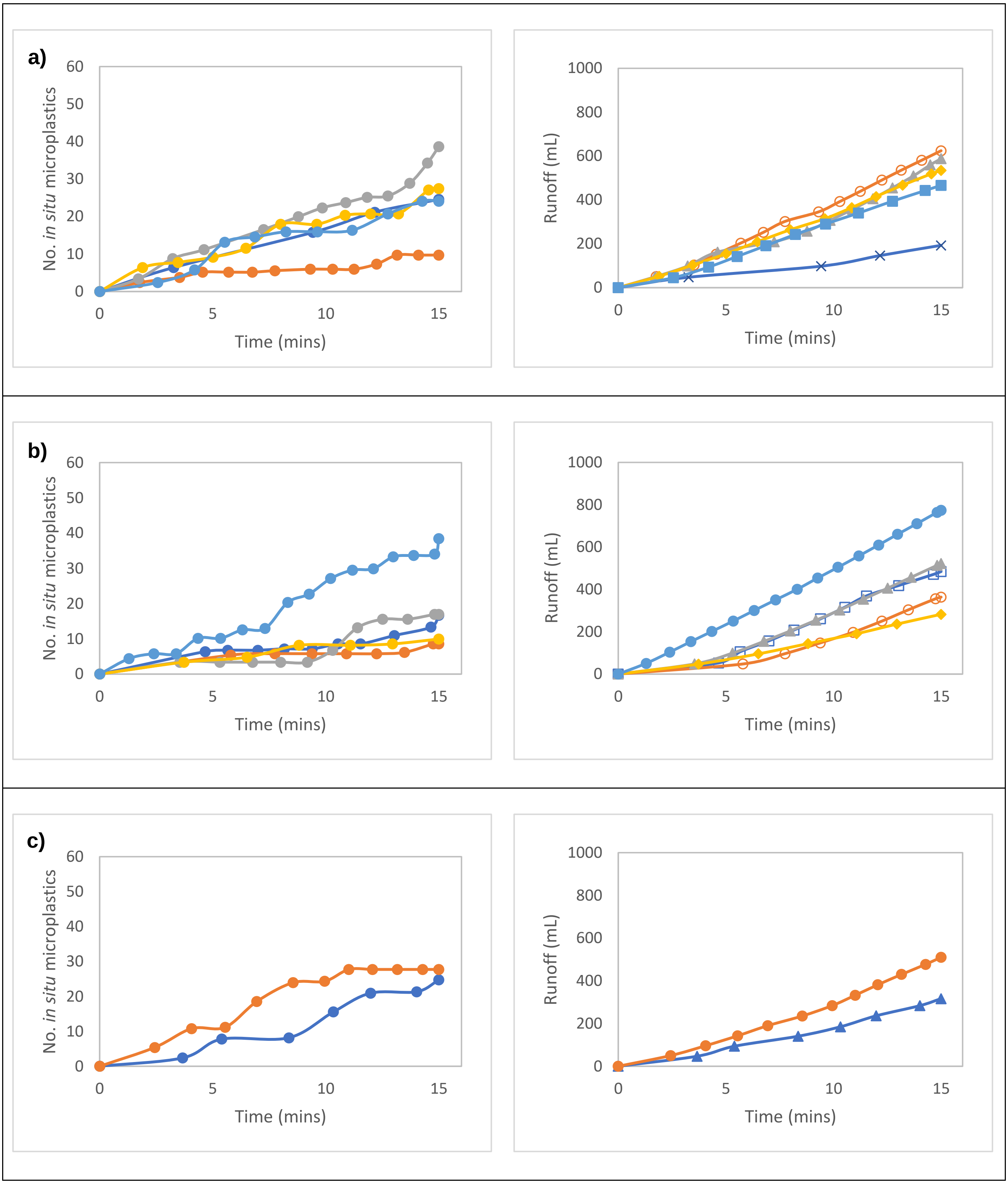

Figure S2a-c: Cumulative *in situ* microplastics and runoff from trail surfaces, including bare, compacted surface on three slopes; a) 0-5°, b) 5-10°, c) 10-15°). Individual lines represent rain simulation on the replicate plots.

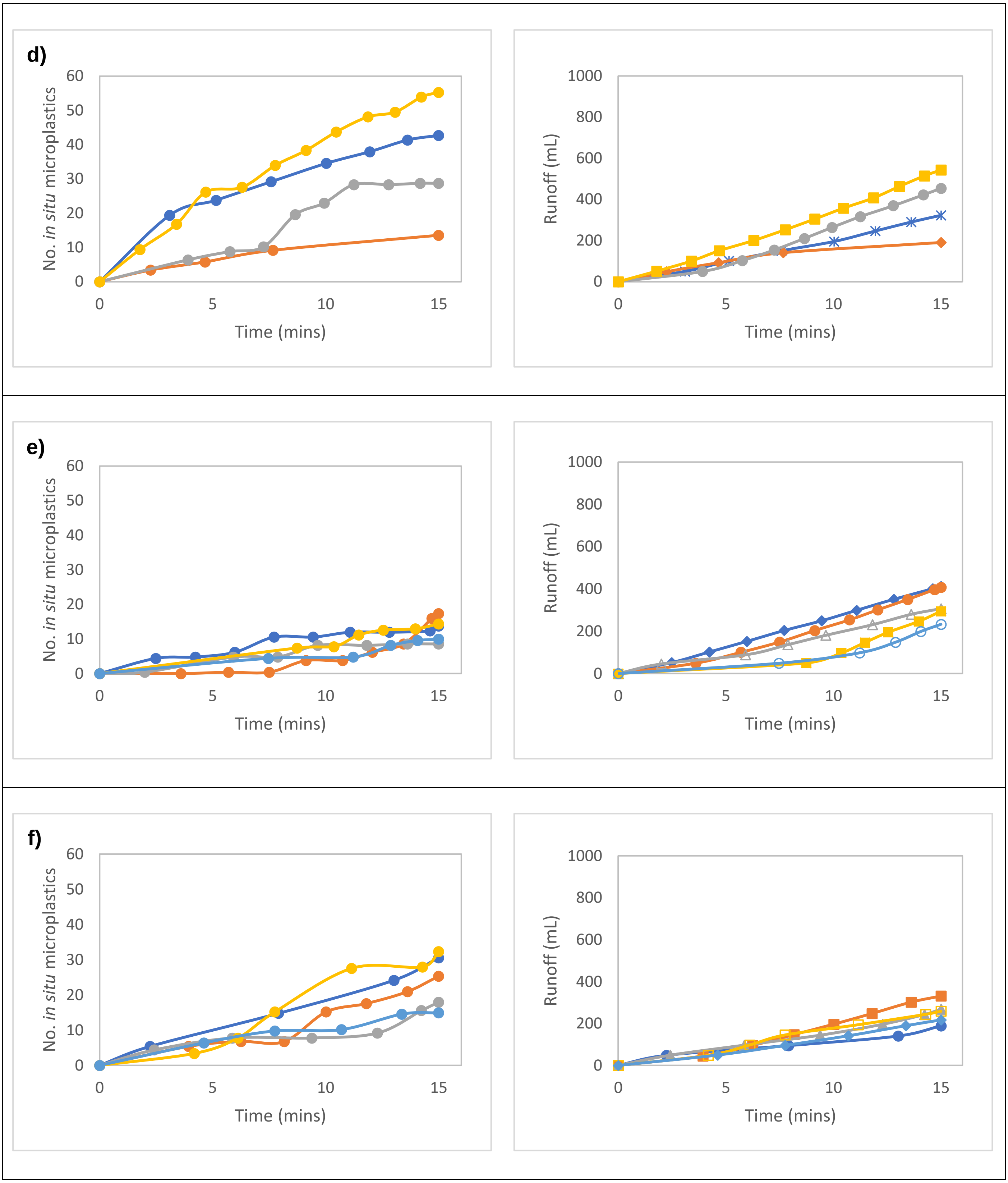

Figure S2d-f: Cumulative *in situ* microplastics and runoff from trail surfaces, including d) vegetated, compacted surface on a low slope (0-5°), e) bare, loose surface on a low slope (0-5°), and f) vegetated, loose surface on a low slope (0-5°). Individual lines represent rain simulation on the replicate plots.

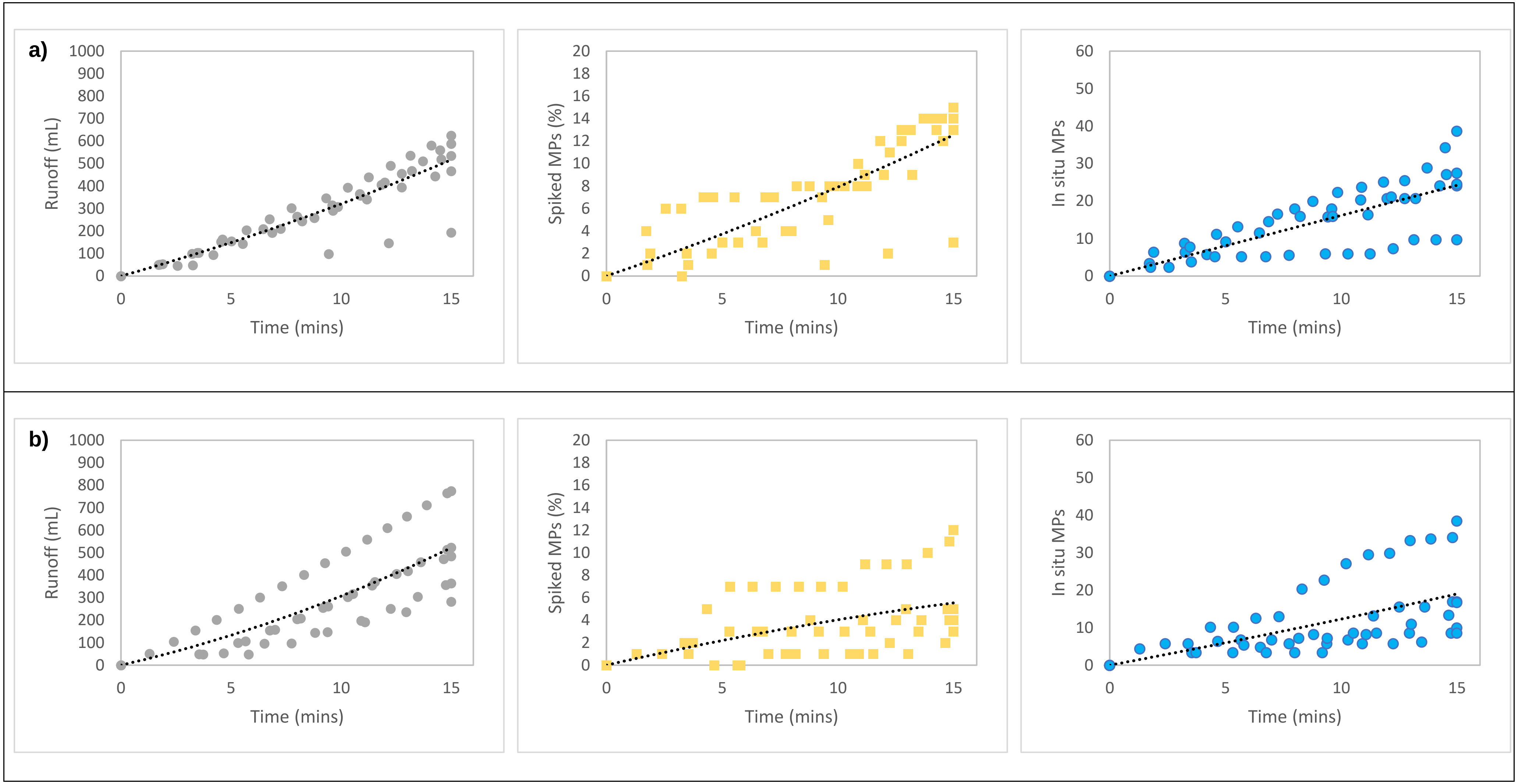

Figure S3a-b: Cumulative runoff, percent spiked microplastics (MPs) and *in situ* MPs from bare, compacted surface trail surfaces on two slopes; a) 0-5°, and b) 5-10°. Dotted lines represent the line of best fit (least squares).

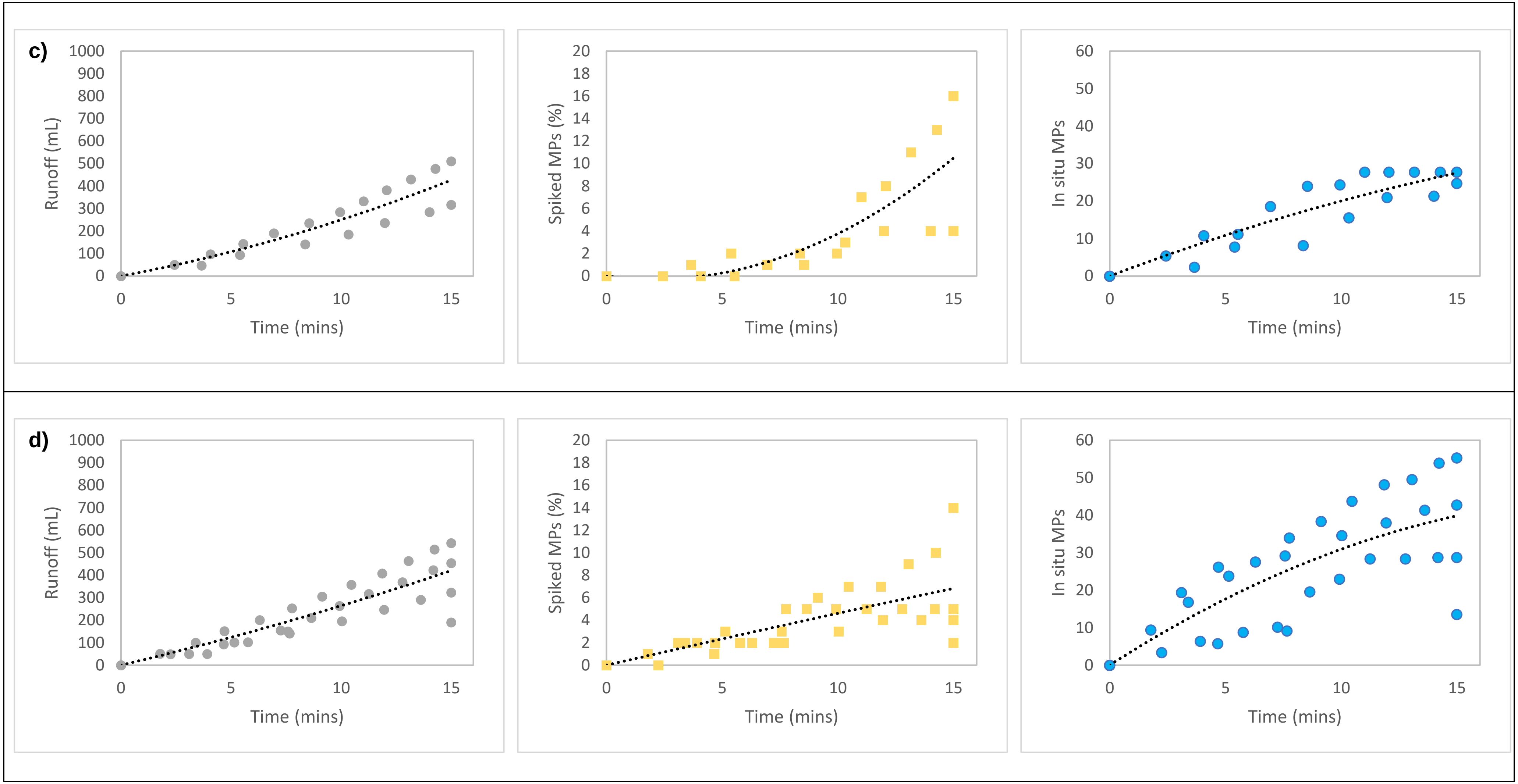

Figure S3c-d: Cumulative runoff, percent spiked microplastics (MPs) and *in situ* MPs from c) bare, compacted surface trail surfaces with a slope 10-15°, and d) vegetated, compacted surface on a low slope (0-5°). Dotted lines represent the line of best fit (least squares).

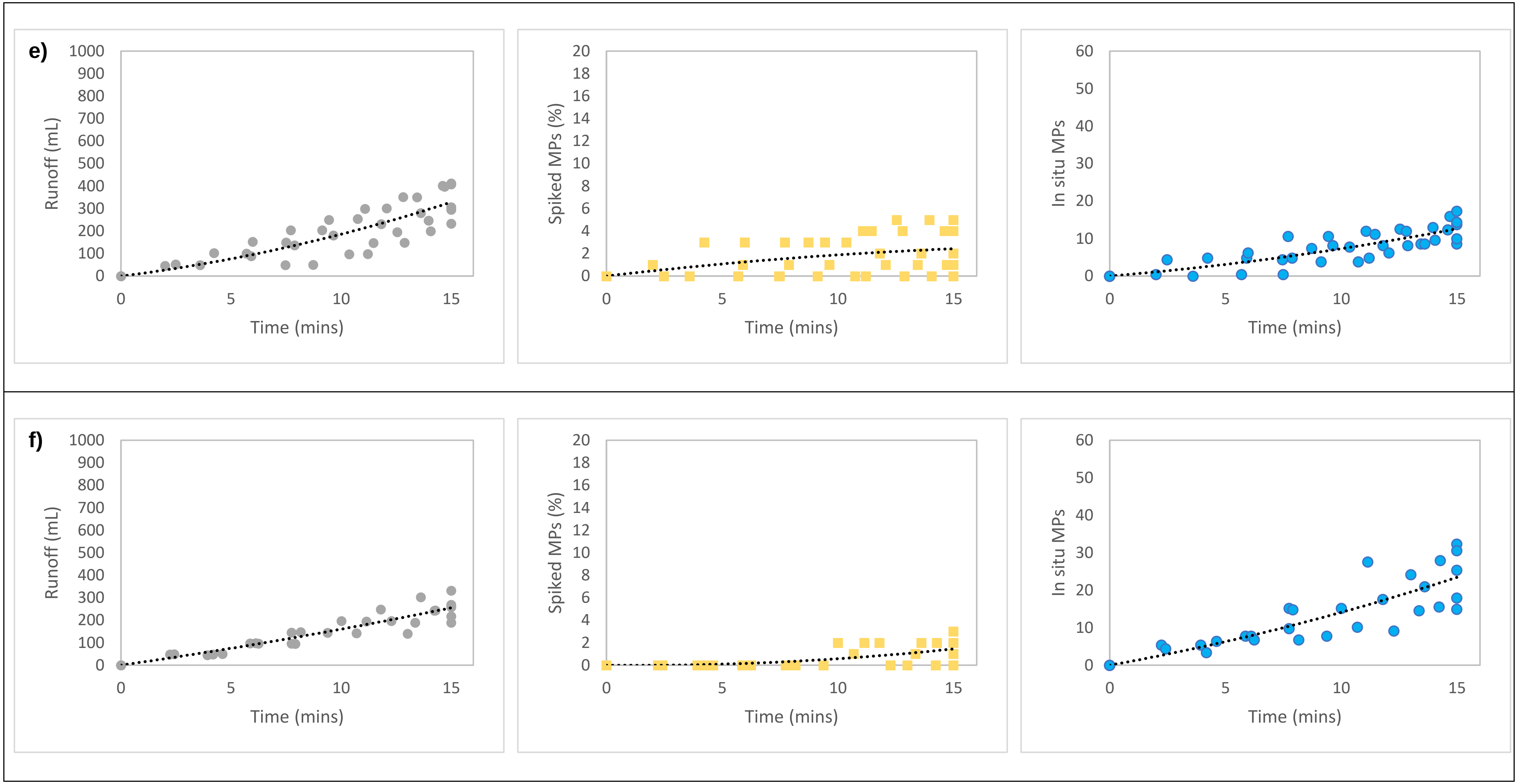

Figure S3e-f: Cumulative runoff, percent spiked microplastics (MPs) and *in situ* MPs from e) bare, loose surface on a low slope (0-5°), and f) vegetated, loose surface on a low slope (0-5°). Dotted lines represent the line of best fit (least squares).
